# Balancing Sensitivity and Specificity Enhances Top and Bottom Ranking in Genomic Prediction of Cultivars

**DOI:** 10.3390/plants14030308

**Published:** 2025-01-21

**Authors:** Osval A. Montesinos-López, Kismiantini, Admas Alemu, Abelardo Montesinos-López, José Cricelio Montesinos-López, Jose Crossa

**Affiliations:** 1 Facultad de Telemática, Universidad de Colima, Colima 28040, Colima, Mexico; 2 Statistics Study Program, Universitas Negeri Yogyakarta, Yogyakarta 55281, Yogyakarta, Indonesia; kismi@uny.ac.id; 3 Department of Plant Breeding, Swedish University of Agricultural Sciences, P.O. Box 101, SE-230 53 Alnarp, Sweden; admas.alemu.abebe@slu.se; 4 Centro Universitario de Ciencias Exactas e Ingenierías (CUCEI), Universidad de Guadalajara, Guadalajara 44430, Jalisco, Mexico; 5 Department of Public Health Sciences, University of California Davis, Davis, CA 95616, USA; 6 Colegio de Postgraduados, Montecillos 56230, Edo. de México, Mexico; 7 International Maize and Wheat Improvement Center (CIMMYT), Carretera Mexico-Veracruz, Km 45, San Pedro Tlaltizapan 52640, Edo. de México, Mexico

**Keywords:** plant breeding, genomics selection, top line selection, bottom line selection, prediction performance

## Abstract

Genomic selection (GS) is a predictive methodology that is revolutionizing plant and animal breeding. However, the practical application of the GS methodology is challenging since a successful implementation requires a good identification of the best lines. For this reason, some approaches have been proposed to be able to select the top (or bottom) lines with more Precision. Despite the varying popularity of methods, with some being notably more efficient than others, this paper delves into the fundamentals of these techniques. We used five models/methods: (1) RC, known as the Bayesian Best Linear Unbiased Predictor (GBLUP); (2) R, which is like RC but uses a threshold; (3) RO, Regression Optimum, that leverages the RC model in its training process to fine-tune the threshold; (4) B, Threshold Bayesian Probit Binary model (TGBLUP) with a threshold of 0.5 to classify the cultivars as top or non-top; (5) BO is the TGBLUP but the threshold used is an optimal probability threshold that guarantees similar Sensitivity and Specificity. We also present a benchmark comparison of existing approaches for selecting the top (or bottom) performers, utilizing five real datasets for comprehensive analysis. For methods that necessitate a rigorous tuning process, we suggest a streamlined tuning approach that significantly decreases implementation time without notably compromising performance. Our analysis revealed that the regression optimal (RO) method outperformed other models across the five real datasets, achieving superior results in terms of the F1 score. Specifically, RO was more effective than models R, B, RC, and BO by 60.87, 42.37, 17.63, and 9.62%, respectively. When looking at the Kappa coefficient, the RO model was better than models B, BO, R, and RC by 37.46, 36.21, 52.18, and 3.95%, respectively. In terms of Sensitivity, the RO model outperformed models B, R, and RC by 145.74, 250.41, and 86.20, respectively. The second-best model was the model BO. It is important to point out that in the first stage, the BO and RO approaches train a classification and regression model, respectively, to classify the lines as the top (bottom) or not the top (not the bottom). However, both the BO and RO approaches optimize a threshold in the second stage to perform the classification of the lines that minimize the difference between the Sensitivity and Specificity. The BO and RO methods are superior for the selection of the top (or bottom) lines. For this reason, we encourage breeders to adopt these approaches to increase genetic gain in plant breeding programs.

## 1. Introduction

The need to produce more food in less arable land arises from several key factors. With a rapidly growing global population, it is essential to increase food production to meet the rising demand. However, the availability of arable land is limited, and various challenges such as urbanization, soil degradation, and climate change are facing it. By maximizing food production on limited land, we can ensure environmental sustainability by minimizing deforestation and reducing the need for chemical inputs. Additionally, producing more food on less land contributes to food security, particularly in regions vulnerable to hunger and malnutrition. It also improves economic efficiency in agriculture, leading to enhanced profitability, rural development, and overall economic growth [1].

Breeding methodologies like genomic selection (GS) play a crucial role in improving productivity worldwide due to several key factors. It enables precision breeding by identifying specific genes and markers associated with desirable traits, allowing for targeted selection of high-yielding varieties or breeds. By predicting genetic potential early on, GS accelerates breeding cycles and expedites the development of productive individuals. The accuracy of trait predictions is enhanced through the utilization of genomic data, enabling informed decisions for selecting individuals with higher productivity traits [2]. GS also facilitates the development of cultivars or breeds that are adaptable to diverse environments, ensuring productivity across different regions [3]. Moreover, by optimizing breeding efforts, GS promotes sustainable resource management, minimizing waste and environmental impact while meeting global food demands.

Genomic selection leverages advances in genomics and data analysis as it allows breeders to make more informed decisions in selecting individuals with desired traits, leading to faster and more precise breeding outcomes. By utilizing large-scale genomic data, GS accurately predicts the genetic potential of plants at an early stage, significantly reducing the time and resources required for traditional breeding methods. This acceleration of breeding cycles enables the development of improved varieties with enhanced traits such as yield, disease resistance, and nutritional quality. Furthermore, GS expands the scope of breeding by identifying and incorporating favorable traits from diverse genetic backgrounds, promoting genetic diversity and adaptability. Overall, GS is transforming plant breeding by optimizing efficiency, precision, and genetic gains, ultimately contributing to the development of sustainable and high-performing crop varieties [3,4].

However, it is important to recognize that the presence of genes or markers alone does not guarantee a high-yielding variety that is well adapted to stress conditions. This limitation arises because not all identified genes are necessarily functional in a given context, particularly under abiotic or biotic stress conditions. To address this, transcriptomics and associative transcriptomics play a crucial role in identifying functional genes linked to specific traits, such as stress tolerance. These approaches enable the assessment of gene expression and functionality, thereby offering deeper insights into which genes are actively contributing to a trait. Incorporating transcriptomic data into genomic prediction models can significantly enhance the precision of selecting stress-tolerant, high-yielding varieties, providing a complementary layer of information beyond marker-based selection [5,6].

Other challenges associated with implementing GS in plant breeding programs are when dealing with large and complex genomes, such as those of wheat and maize. Large plant genomes often contain a substantial amount of repetitive DNA, which can complicate marker identification and analysis [7]. Additionally, the lack of comprehensive knowledge about the genetic basis of many important agronomic traits further complicates the application of GS, particularly for polygenic traits that are influenced by numerous loci with small effects [8].

Furthermore, genes may behave differently depending on the genetic context in which they are expressed, leading to variable performance across environments and genetic populations [9]. The lack of robust, high-throughput phenotyping platforms capable of capturing complex traits across environments remains a significant bottleneck in breeding programs [10]. Addressing these limitations requires further integration of functional genomics, high-throughput phenotyping, and better models that account for environmental and genetic interactions. In summary, these points enrich the narrative with supporting citations while addressing the challenges of genomic selection, such as large genomes, insufficient phenotyping, and gene expression variability.

However, for a successful implementation of GS in breeding programs, high accuracy is essential [11]. Accurate predictions enable breeders to make reliable decisions, maximizing the genetic potential of offspring and leading to improved traits and higher productivity. It helps avoid selecting individuals that are not the best, saving valuable resources and time. High accuracy allows breeders to focus on specific traits of interest, meeting market demands and environmental challenges. Trust and confidence in the approach are built through accurate predictions, facilitating wider adoption of GS. Ultimately, accurate predictions drive genetic improvement in crops and livestock, contributing to the success of breeding programs.

To enhance the efficiency of the GS methodology, a wide array of statistical machine learning models has been explored, encompassing both parametric approaches such as mixed models, Bayesian models, and penalized regression, as well as nonparametric models such as random forest, gradient boosting machine, and deep learning [12]. Nevertheless, it is essential to recognize the constraints imposed by the No Free Lunch Theorem in statistical machine learning, which asserts the absence of a universal algorithm excelling in all conceivable tasks. Consequently, any enhancement in performance for one task necessitates a trade-off, potentially resulting in reduced performance in another domain. This underscores the absence of one-size-fits-all solutions in the pursuit of optimal performance across diverse problem domains. As a result, even though certain statistical machine learning models have exhibited commendable performance within the genomic prediction context, practical implementation often encounters challenges due to insufficient prediction accuracies. This limitation can be attributed to the multifaceted nature of the GS methodology, a predictive approach influenced by numerous factors.

In the realm of genomic selection (GS), where the primary objective is to identify and select the most promising genetic lines with high accuracy, it becomes imperative to incorporate robust metrics into the selection process. Sensitivity and Specificity serve as pivotal measures in this regard. Sensitivity, traditionally applied in diagnostic testing contexts, signifies the capacity of a test to accurately identify individuals possessing a particular characteristic. Sensitivity delineates the effectiveness of a test in correctly pinpointing individuals who exhibit the desired characteristic [13]. A heightened Sensitivity translates to a diminished rate of false negatives, ensuring that fewer instances of the desired characteristic are overlooked or misjudged by the selection process. In the context of plant breeding, where the aim is to pinpoint the most advantageous genetic lines, it is paramount for the models to exhibit a commendable level of Sensitivity. This ensures that the selection process accurately identifies and advances the most promising genetic lines, thereby optimizing breeding outcomes.

Conversely, Specificity characterizes the capability of a diagnostic test to accurately exclude individuals lacking a specific characteristic. It quantifies the precision with which the test identifies individuals devoid of the characteristic under consideration [13]. Within the realm of plant breeding, it is equally crucial to uphold a reasonable level of Specificity. This ensures that the selection process does not erroneously favor or advance genetic lines that do not possess the desired characteristics. By maintaining a balanced approach that encompasses both Sensitivity and Specificity, the selection process can effectively discriminate between superior genetic lines and those that do not meet the desired criteria, thereby enhancing the efficiency and efficacy of genomic selection in plant breeding endeavors. However, since the selection of the best lines in the context of GS is performed with predictions resulting from regression models, selecting those lines with larger predicted phenotypic or breeding values when the trait of interest is grain yield or other traits where the larger score in the trait is better.

On the other hand, lower predicted values can often be desired because they signify a higher probability of desirable traits (disease resistance) in the selected lines. Therefore, in genomic prediction for traits such as disease resistance or pest tolerance, where lower values indicate superior performance, selecting lines with lower predicted values can lead to the development of improved crop varieties with enhanced resilience to biotic stresses. Under both scenarios of selecting predicted lines since the lines with larger (or lower) predictions, the resulting selection guarantees considerably high Specificity and considerably low Sensitivity. However, many plant breeders may not be fully aware of this issue, as Sensitivity and Specificity metrics are not commonly integrated explicitly into their selection processes for identifying the best genetic lines. However, it is important to recognize that Sensitivity and Specificity are both critical measures of the accuracy and reliability of selected lines in plant breeding programs.

For this reason, a balanced good level of Sensitivity and Specificity is desired in plant breeding since high Sensitivity ensures that valuable cultivars with desirable characteristics are not overlooked, thus maximizing the potential for identifying superior genetic material. Conversely, high Specificity ensures that resources are efficiently allocated by effectively excluding cultivars lacking the desired characteristics. For this reason, in this paper, we explore some existing methods for selecting the best lines and we evaluate their Sensitivity and Specificity. In this research, we aim to provide detailed explanations of each existing selection method, introduce Sensitivity and Specificity as metrics for comparing the chosen selection candidates, and conduct benchmarking to enhance empirical evidence of their performance. Our benchmarking analysis encompasses five real datasets, comprising four related to maize and one to soybean, each with multiple traits and environments.

## 2. Materials and Methods

This study included Maize Data 2–5 (Maize_1. Maize_2, Maize_3, Maize_4) and Soybean 9 (Soybean_4) from the data employed by Montesinos-Lopez et al. [14] (see Table A1 from Montesinos-Lopez et al. [14]).

The current analysis with different models is based on the various datasets described in Table 1. Four datasets are from maize trials while the remaining are from soybean. The population size of datasets ranged from 1864 to 999 genotypes while the number of applied quality SNP markers ranged from 1803 to 4085. The number of environments for the four maize datasets was 11 while the remaining soybean dataset had 8 environments. Four traits were included on the maize datasets while the soybean dataset comprised six traits.

**Datasets**

**Table 1:** Description of the datasets used for performing the benchmarking analysis. Data comes from Alencar Xavier. et al. (2021) [15], recently cited by Montesinos-Lopez et al. 2024 [14].

Data Number	Dataset	Genotypes	Markers	Environments	Traits	Reference
Data 1	Maize_1	1000	4085	11	4	Maize Data 2 from [14,15]
Data 2	Maize_2	1000	4085	11	4	Maize Data 3 from [14,15]
Data 3	Maize_3	1000	4085	11	4	Maize Data 4 from [14,15]
Data 4	Maize_4	999	4085	11	4	Maize Data 5 from [14,15]
Data 5	Soybean_4	1864	1803	8	6	Soybean Data 9 from [14,15]

All statistical models provided in the next section were implemented for each environment of each dataset and the results were reported for each dataset across the environment to summarize the results. For this reason, the statistical models given in the next section of the predictor do not take the effect of environments into account.

### 2.1. Statistical Models

The 5 statistical models studied in this research can be grouped into 2 main classes:

1. GBLUP (RC), GBLUP with a threshold (R), and GBLUP with an optimal fine-tuned threshold (RO). As described below, models R and RO are the basic RC models but with certain refinements on how the thresholds are defined;
2. TGBLUP (threshold GBLUP) with a threshold = 0.5 that classifies candidates as top and non-top (B) and a TBLUP with an optimal probability threshold is denoted as BO model. The BO model is also trained with the TGBLUP but in place of using a threshold of 0.5 to classify the lines as top and not top, an optimal probability threshold that guarantees similar Sensitivity and Specificity is used.

Thus, our study includes models RC, R, and RO, as well as B and BO that are described below.

#### 2.1.1. Model RC

Model RC, known as the Bayesian Best Linear Unbiased Predictor (GBLUP) model, is structured as a regression framework. The model is defined as follows:

(1) $$Y_{i}=\mu +g_{i}+\epsilon_{i}$$

Here, $Y_{i}$ represents the continuous response variable observed in the ith instance. $Y_{i}$ are BLUEs resulting after accounting for environments and experimental factors (blocks, reps, etc). μ stands for the general mean or intercept. $g_{j},$ i=1,…,J, signifies the random effect associated with the ith genotype. Additionally, $\epsilon_{i}$ denotes the random error component for the ith genotype, distributed as an independent normal random variable with a mean of 0 and a variance of $\sigma^{2}$. It is assumed that $g={\left(g_{1},\ldots ,g_{J}\right)}^{T}∼N_{J}\left(0,\sigma_{g}^{2}G\right)$, where G is a linear kernel referred to as the genomic relationship matrix, calculated using the method outlined in [16]. This model has been implemented in the R statistical software [17], utilizing the BGLR library [18]. 

For each fold of the cross-validation, we train the model (1) using the whole training information, and the predictions are performed for the whole testing set and then the predicted values are classified as top lines or not top lines. Given our interest in selecting the top-performing lines for each trait, we introduce the threshold $Y_{\tau }$. This threshold is determined by the empirical quantile τ of training response values ($Y_{1}$, …, $Y_{n_{tr}}$). For our purposes, we have chosen τ=0.8, but it is important to note that any other value between 0 and 1 can be employed. The classification of the lines in the testing set as top lines (1) and not top lines (0) under this model was performed by first ordering the lines in the testing set (of both observed and predicted) in decreasing order. Then, we identified how many lines of the observed response were larger than the threshold $Y_{\tau }$, and then this same number of lines was selected from the ordered vector of predicted lines. It is important to point out that, from the predicted lines, we selected an equal number of lines as in the vector of the observed response variable that had the best performance, that is, the top lines, but in this case, they were not chosen regarding of the threshold $Y_{\tau }$. Next the lines that matched between the two selected vectors were classified as top lines and those that did not were classified as not top lines.

#### 2.1.2. Model R

Under this model, the predictions were obtained exactly with the trained model RC given above with Equation (1), but the process of classification was performed differently. For this reason, model R stood for GBLUP with a threshold. Here, the threshold, $Y_{\tau }$, was employed to classify the lines into two categories: top lines (denoted as 1) if $\hat{Y_{i}}>Y_{\tau }$, for $i=1,\ldots ,n_{tst}$) and not top lines (denotes as 0) if $\hat{Y_{i}}<Y_{\tau },\;\mathrm{for}\;i=1,\ldots ,n_{tst}$. That is, under this approach after obtaining the continuous predictions using the model RC, for any lines with predicted values exceeding the threshold, $Y_{\tau },\;$ were classified as top lines, while those with predicted values below the threshold were classified as not top lines [19]. 

#### 2.1.3. Model RO

**Figure 1:**
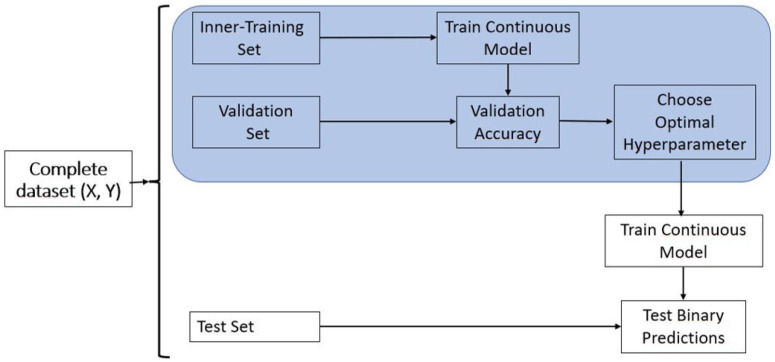
Schematic depiction of the model RO training process. **X** represents the input data, encompassing markers and other covariates, while **Y** signifies the continuous response variable. The final predictions are binary, with a value of 1 indicating top lines and 0 representing not top lines.

The acronym RO stands for Regression Optimum, and this model leverages the RC model in its training process to fine-tune the threshold. For this reason, this model consists of the GBLUP model with an optimal threshold. Also, here, the initial threshold was the 80% quantile of the response variable from the training set. However, this initial threshold was adjusted to ensure a similar Sensitivity and Specificity. A schematic representation of the procedural steps involved in the training process of Model RO is illustrated in Figure 1. According to the previously applied approach by Montesinos-López et al. [19], these steps are briefly elucidated as follows:

Step 1: Initiate by partitioning the data into distinct subsets, comprising the inner-training, validation, and test sets;

Step 2: Proceed to train Model RC using the inner-training set while utilizing the original response variable;

Step 3: Utilize the trained Model RC (from Step 2) on the validation set to compute predicted continuous values, ${\hat{Y}}_{Val,l}$ for $i=1,\ldots ,n_{val}$. Subsequently, employ these predicted values to look for the optimal threshold $Y_{o}$;

Step 4: This optimal threshold ($Y_{o}$) is identified such that it guarantees that minimizes the average squared difference between Sensitivity and Specificity;

Step 5: Next, with the complete training dataset (comprising both the inner-training and validation sets), retrain Model RC. Use this refitted model to compute predicted values for the testing set, resulting in ${\hat{Y}}_{Test,l}$ for $i=1,\ldots ,n_{Test}$;

Step 6: Subsequently, employing the optimal threshold ($Y_{o})$ computed in Step 4 and the predicted values from the testing set in Step 5, classify the lines. If ${\hat{Y}}_{Test,l}>Y_{o}$, categorize the line as a top line (1); otherwise, classify it as a not top line (0).

Figure 1 visually illustrates the incorporation of model R within the training process of model RO.

It is noteworthy that this refined optimal rule can be expressed in terms of conventional threshold values ($Y_{\tau }$). This equivalence arises from the classification of a line as top $\left(\hat{Y_{i}}>Y_{o}\right)$ being analogous to categorizing it if ${\hat{Y}}_{i}^{*}>Y_{\tau }$ where ${\hat{Y}}_{i}^{*}=\hat{Y_{i}}(Y_{\tau }/Y_{o})$. These modified predicted values, or adjusted predicted values, are designed to ensure a congruent balance between Sensitivity and Specificity.

It is essential to highlight that, for this RO method, we have introduced a more computationally efficient version, which we refer to as the “Simple (S) RO method”. This approach involves training only one time in place of k times model (1) using the complete training (inner-training + validation) dataset and then using this trained model to predict the complete testing sets. This means that we utilize the predictions from model RC prior to classification and for this reason; this S RO method is computationally more efficient since only one time is trained the method RC. Subsequently, with the results obtained from the predicted values we divide these predicted values into an outer training and validation set. This splitting process of the results of the S RO method is carried out for choosing the optimal threshold but uses the predicted values resulting in training only one time the full training set. Employing a 10-fold inner cross-validation, we determine the optimal threshold for classifying the lines in the testing dataset. The schematic representation of this Simple RO method is akin to Figure 1, with the noteworthy difference being that we only train model (1) once. This eliminates the need to repeatedly train the model (1) for the number of folds specified in the inner cross-validation, resulting in a significantly more computationally efficient implementation of the Simple RO method. In all models under study, we focus on the selection process of selecting the top-performing lines, assuming that these are the best lines. For this reason, Sensitivity will be associated with how these top lines were selected while Specificity regards how the non-top lines are not selected.

#### 2.1.4. Model B

Model B, known as the Threshold Bayesian Probit Binary model (TGBLUP), operates on the premise that given $g_{i}$ (covariates of dimension J), $Y_{bi}$ is a random variable taking binary values, 0 and 1, with the following probabilities:

(2) $$P\left(Y_{bi}=1|g_{i}\right)=\Phi \left(\beta_{0}+g_{i}\right)=P\left(l_{i}>0\right)$$

where $\;\beta_{0}$ represents the intercept parameter, $g_{i}$ signifies the random effect associated with the ith genotype, distributed as per the definition in model (1). Φ is the cumulative distribution function of the standard normal distribution. Furthermore, $l_{i}=\beta_{0}+g_{i}{+\epsilon }_{i}$ represents the latent continuous normal process that underlies the observed categories (top lines and not top lines), where $\epsilon_{i}$ is a normal random variable for errors with a mean of 0 and a variance of 1. These $l_{i}$ values are referred to as “liabilities” [20,21]. The binary categorical phenotypes in model (2) are derived from the underlying phenotypic values, $l_{i}$, as follows: $y_{bi}=0$ if $-\infty {<l}_{i}<0$, otherwise $y_{bi}=1.$ Since model (2) is articulated within a Bayesian framework, it assumes a flat prior distribution for $\beta_{0}$ ($f\left(\beta_{0}\right)\propto 1$). The TGBLUP has been implemented in the BGLR package [18] within the R statistical software [17]. Under this model after the training process has been computed the probability of $P\left(Y_{bi}=1|g_{i}\right)$ for each line in the testing set and, if this probability is larger than 0.5, the line is classified as top line; otherwise, the line is classified as not top line.

#### 2.1.5. Model BO

Model BO is also trained with the TGBLUP using the model given in Equation (2), but in place of using a threshold of 0.5 to classify the lines as top and not top, we used an optimal probability threshold that guarantees similar Sensitivity and Specificity. For estimating this optimal probability threshold (hyperparameter), in addition to dividing the data into training and testing, the training set was divided into inner-training and validation. According to Montesinos-López et al. [19], all the steps for implementing this method are given next:

Step 1: Commence by converting the continuous response variable into a binary response variable, utilizing the same threshold, $Y_{\tau },$ as employed previously using the quantile 80% of the training set. Specifically, when the values of the continuous traits surpass the designated threshold, assign them a value of one (1, denoting a top line); otherwise, assign zero (0, signifying not top lines);

Step 2: Initially, partition the data into distinct subsets, namely the inner-training, validation, and test sets;

Step 3: Proceed to train model B, a classification model, using the inner-training set;

Step 4: Employ the trained model B (from Step 3) on the validation set to compute the predicted probabilities, ${\hat{P}}_{Val,l}$ for $i=1,\ldots ,n_{val}$. Subsequently, utilize these predicted probability values to estimate classification accuracy metrics, facilitating the selection of the optimal probability threshold, ${(\tau }_{0})$;

Step 5: Identify the optimal probability threshold, ${(\tau }_{0})$, which minimizes the average of the squared difference between Sensitivity and Specificity;

Step 6: Next, with the complete training dataset (comprising both the inner-training and validation sets), retrain model B and generate probability predictions for the testing set. These results are ${\hat{P}}_{Test,l}$ for $i=1,\ldots ,n_{Test},\;$ within the testing set;

Step 7: Subsequently, employing the optimal probability threshold ($\tau_{0}$) determined in Step 5 and the predicted probabilities from the testing set in Step 6, classify the lines. If ${\hat{P}}_{Test,l}>\tau_{0}$, categorize the line as a top line (1); otherwise, classify it as a not top line (0).

**Figure 2:**
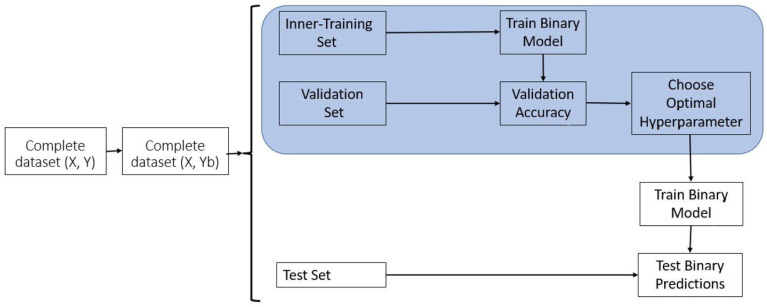
Schematic representation of the training process under model BO. **X** represents the input data, encompassing markers and additional covariates. Meanwhile, Y symbolizes the continuous response variable, and **Y**b signifies the binary response variable generated through the transformation of Y into a binary format. In the ultimate classification, top lines are designated as “1”, whereas not top lines are denoted as “0”.

For further elaboration on these steps, please refer to Figure 2.

In this specific BO method, we have introduced a more computationally efficient version, which we call the “Simple (S) BO method”. Here, is how it works: We train model (2) using the entire training (inner-training + validation) dataset and then use this trained model to predict the entire dataset, leveraging the predictions from model B before classification. This S BO method reduces computational resources since in place of training k-times the model with the training data is trained only one time using the whole training set because the classification model to compute the probabilities is trained only one time in Figure 2. Next, we take the results from these predicted values for each fold and split them into an outer training and validation set. Through a 10-fold inner cross-validation process, we determine the best threshold for classifying the lines in the testing dataset. The visual representation of the Simple BO method is similar to Figure 2, but the key difference is that we only train model (2) once. This eliminates the need to repeatedly train the model (2) for the specified number of folds in the inner cross-validation, resulting in a significantly more efficient implementation of the Simple BO method.

Ultimately, as all seven evaluated models (RC, R, RO, Simple RO, B, BO, and Simple BO) generate predictions in the form of binary outcomes (0 for not top lines and 1 for top lines), classification metrics have been computed to evaluate prediction accuracy for the testing sets.

### 2.2. Evaluation of Prediction Performance

In our study, we conducted a rigorous evaluation process for benchmarking the proposed models using a nested cross-validation approach. This approach involved two levels of cross-validation: outer-fold cross-validation and inner-fold cross-validation, as outlined in Montesinos López et al. [12] The outer-fold cross-validation aimed to assess the prediction accuracy of our models on unseen data. We utilized a 5-fold cross-validation strategy, where the dataset was randomly split into five subsets or “folds”. The model was trained on four of these folds while the remaining one was reserved for testing. This process was repeated until each fold had served as the test set once. It is important to note that the test sets were exclusively used for evaluation purposes and were never incorporated into the model training process. The average performance across these 5 testing sets was reported, employing four distinct metrics, as elaborated in the subsequent sections.

Additionally, we computed prediction performance metrics based on the average results obtained from the 5-fold cross-validation. These metrics included the Kappa coefficient, Sensitivity, Specificity, and F1 score. For models B, R, and RC, no hyperparameter tuning was necessary, but for models BO and RO, we fine-tuned a critical hyperparameter: the probability threshold for model BO and a threshold for the RO model. This was carried out to ensure a balance between Sensitivity and Specificity. To achieve this balance, we conducted inner-fold cross-validation using ten-folds. The goal was to optimize the threshold values, which were selected as the average threshold value across the ten-folds of the inner cross-validation. These optimized thresholds were subsequently employed in the classification of lines into top and non-top categories within each testing set, as illustrated in Figure 1 and Figure 2.

Subsequently, we computed various metrics based on the predictions generated by three distinct models (R, RC, RO, B, and BO) for each testing dataset. These metrics are elucidated as follows, Kappa Coefficient (κ): The Kappa coefficient is a statistical measure used to assess the degree of agreement among raters, accounting for chance. It is defined as: $\kappa =\frac{P_{0}-P_{e}}{1-P_{e}}$, Where $P_{0}\;$ represents the agreement between the predicted and observed values and is computed as (TP + TN)/N, where TN denotes the number of true negatives, TP signifies the number of true positives, FN represents the number of false negatives, FP indicates the number of false positives, and N is defined as N = TP + TN + FP + FN. $P_{e}$ denotes the probability of agreement and is calculated as $P_{e}$ = (TP + FN)/N × (TP + FP)/N + (FP + TN)/N × (FN + TN)/N. Sensitivity: Sensitivity is the probability of obtaining a positive test result when the true condition is indeed positive. It is expressed as: Sensitivity = TP/(TP + FN). Specificity: Specificity represents the probability of obtaining a negative test result when the true condition is negative. It is formulated as: Specificity = TN/(TN + FP). Precision: Precision measures the ratio of correctly predicted positive observations to the total predicted positive observations and is defined as: Precision = TP/(TP + FP). A higher Precision value corresponds to a lower false positive rate and, importantly, signifies superior prediction accuracy. These metrics serve as critical indicators for evaluating the performance of our models, providing valuable insights into their predictive capabilities and the accuracy of their assessments.

The F1 Score, as a composite metric, provides a balanced evaluation by considering both Sensitivity and Precision. This holistic approach acknowledges the impact of false negatives and false positives in the assessment, making it particularly valuable when dealing with datasets that exhibit imbalanced class distribution. Accuracy, on the other hand, is most effective when the costs associated with false positives and false negatives are comparable. If there is a significant disparity in the cost implications of these errors, it is advisable to examine both Precision and Sensitivity [13].

To facilitate interpretation, we compared model RO vs. R, RC, B, and BO and model BO vs. R, RC, B, and RO. We computed the relative efficiencies (RE) in terms of the Kappa score, denoted as $RE_{Kappa}$. This calculation is expressed as follows:

$$RE_{Kappa}=\frac{{Kappa}_{y}}{{Kappa}_{z}}$$

Here, ${Kappa}_{y}$ and ${Kappa}_{z}$ represent the Kappa coefficients of one of the five models (RO, R, RC, B, and BO). Similarly, concerning Sensitivity, the relative efficiency (RE) denoted as $RE_{Sensitivity}$, was computed as:

$$RE_{Sensitivity}=\frac{{Sensitivity}_{y}}{{Sensitivity}_{z}}$$

Again, this calculation involved any of the five models. The same approach was employed to calculate the relative efficiency for the F1 score and Specificity. Under all four metrics (Kappa, Sensitivity, Specificity, F1), if $RE_{x}>1$, where x represents Kappa, Sensitivity, Specificity, or F1, indicates that the method y yielded superior prediction performance. Conversely, when $RE_{x}<1$, the preferable method is z. In cases where $RE_{x}=1$, both methods exhibit equal efficiency in their predictive capabilities. This systematic evaluation approach aids in identifying the most effective method for the given context.

## 3. Results

The results of the genomic prediction models are extensive and include detailed predictions across a range of environments and traits. While the statistical analysis is rigorous, it is important to acknowledge that without a clear understanding of the original data sources or specific traits, the predictions may seem complex or difficult to interpret from a practical breeding perspective. However, to address this concern, we have validated the model predictions against observed phenotypic data to ensure their relevance.

By comparing the genomic estimated breeding values (GEBVs) with real-world field performance, we can confirm that the predictions correspond well to actual breeding outcomes. This validation reinforces the utility of the models, moving beyond a theoretical exercise and demonstrating their applicability in guiding the selection of top-ranking cultivars for breeding programs. Thus, while the statistical framework is detailed, the practical value of these predictions is rooted in their strong correspondence to reality, making them a valuable tool for enhancing selection efficiency in plant breeding.

The results are given in five sections. Section 1, Section 2, Section 3 and Section 4 present the prediction performance of datasets: Maize_1, Maize_2, Maize_3 and Soybean, while Section 5 provides a summary of the prediction performance across all datasets. Moreover, the results for the dataset Maize_4 are provided in Appendix B.

### 3.1. Maize_1 Data

**Figure 3:**
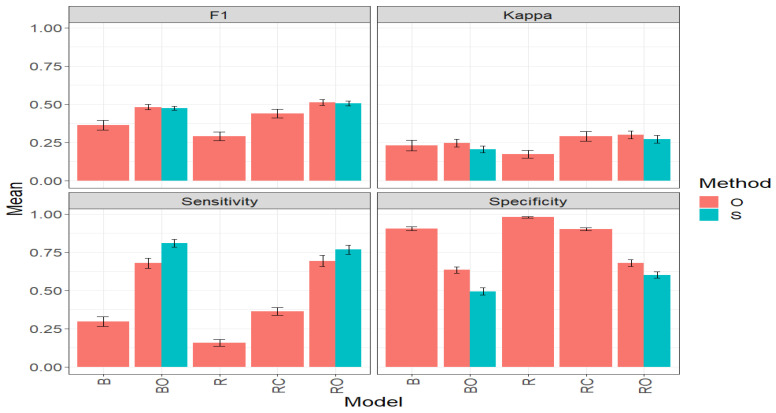
Prediction performance for dataset Maize_1 using original (O) and simplified (S) methods. The results are presented for models B, BO, R, RC, and RO in terms of the metrics: F1 score, Kappa coefficient, Sensitivity, and Specificity.

Figure 3 and Table A1 (Appendix A) present the results for the Maize_1 dataset from the evaluation of two methods for selecting the best candidate lines in a genetic improvement program, the simplified method (S), and the original method (O) proposed by Montesinos-López et al. [12]. The results in this table are presented for five models: Binomial (B), Optimized Binomial (BO), conventional regression with a threshold (R), threshold-free conventional regression (RC), and optimized regression (RO), under four classification metrics: F1 score, Kappa coefficient, Sensitivity, and Specificity. The reported values are the mean across traits and environments. For each of these metrics, the higher the mean value and the lower the standard error (SE), the better the results obtained by each model and, consequently, by each method.

#### 3.1.1. F1 Score

Under the F1 Score metric, concerning the “O” method, the best-performing model in terms of the average F1 score value was the RO model, with an average value of 0.5126. The second-best model was BO with an average value of 0.4813, representing a significant improvement of 32.30% compared to the B model, which had an average value of 0.3638 (See Figure 3 and Table A1). However, BO was outperformed by RO by 6.50%. The worst performance was observed in the R model, with an average value of 0.2895, representing a decrease of 39.84% compared to BO and 43.52% compared to RO. These results can be observed graphically in Figure 3.

When comparing the “O” and “S” methods using the BO and RO models, it is observed that, in both cases, the “O” method slightly outperforms the “S” method in terms of the F1 Score. For BO, the average F1 Score value in the “O” method (0.4813) is 1.56% higher than in the “S” method (0.4739). For RO, the average F1 Score value in the “O” method (0.5126) is 1.39% higher than in the “S” method (0.5056). These results reflect that the “O” method has a slight advantage over the “S” method in terms of accuracy in selecting the top lines; however, the “S” method is considerably less computationally demanding but sacrifices some accuracy.

#### 3.1.2. Kappa Coefficient

The results under the Kappa metric, in the context of the “O” method, are detailed in Table A1 and Figure 3. The RO model stands out as the best, with an average Kappa value of 0.3004. It is followed by the RC model with an average value of 0.2914, and then BO, B, and R in that order. RO outperforms BO (average value of 0.2462) by 22%. In contrast, the R model shows the worst performance, with an average Kappa value of 0.1734, marking a significant decrease of 29.58% compared to BO and 42.28% compared to RO (see Figure 3 and Table 1).

On the other hand, when comparing the “O” and “S” methods in terms of the average results of BO and RO, it can be observed in Table A1 and Figure 3 that the “O” method outperformed the “S” method. For O, the average Kappa value of BO (0.2462) is 20.26% higher than the value achieved by the “S” method (BO = 0.2048). In the case of RO, the average Kappa value in the “O” method is 0.3004, which is 10.66% higher than that of the “S” method (0.2715). However, it is essential to remember that the “S” method offers a considerable advantage in computational efficiency in its operation compared to the “O” method.

#### 3.1.3. Sensitivity

In the Maize_1 dataset, five models (B, BO, R, RC, and RO) were evaluated under the “O” and “S” methods in terms of the Sensitivity metric, and the results are shown in Table 1. This metric is essential for evaluating a model’s ability to correctly identify “top lines when they are actually top”, thus complementing the information provided by the F1 and Kappa metrics that have also been previously analyzed in the same context. Figure 3 provides a graphical representation of the results of this evaluation.

In the “O” method, the RO model stands out by obtaining the highest average value of Sensitivity, with an impressive 0.6936. It is closely followed by the BO model with an average value of 0.6784, representing a significant increase of 127.54% compared to the B model, which has an average value of 0.2982. In contrast, the R model shows the lowest performance with an average Sensitivity of 0.1586, demonstrating a challenge in its ability to identify “top lines”.

Comparing the “O” and “S” methods directly using the BO and RO models, it is observed that, in both cases, the “S” method strongly outperforms the “O” method in terms of Sensitivity. For BO, the average Sensitivity value in the “S” method (0.8091) is 19.3% higher than in the “O” method (0.6784). Likewise, for RO, the average Sensitivity value in the “S” method (0.7673) is 10.62% higher than in the “O” method (0.6936).

#### 3.1.4. Specificity

In the “O” method, the R model stands out by achieving the highest average value of Specificity (0.9786), indicating a strong ability to identify the “non-top” lines. It is closely followed by the B and RC models with average values of 0.9036 and 0.9017, respectively. On the other hand, the RO and BO models show the lowest performances, with an average Specificity of 0.6797 and 0.6340, respectively. This indicates, for example, that BO has a 35.22% lower ability to effectively identify the non-top lines compared to the R model.

When comparing the “O” and “S” methods directly using the BO and RO models, it is observed that, in both cases, the “O” method surpasses the “S” method in terms of a higher Specificity value. For the BO model, the average Specificity value in the “O” method (0.6339) is 28.4% higher than in the “S” method (0.4939). For RO, the average Specificity value in the “O” method (0.6797) is 13.0% higher than in the “S” method (0.6016). Consequently, and bearing in mind that a very high value of Specificity is not necessarily the best, as it affects Sensitivity, the S method exhibits better behavior.

In conclusion, this exhaustive study of the “O” and “S” methods applied to the Maize_1 dataset, evaluating crucial metrics such as F1, Kappa, Sensitivity, and Specificity, reveals clear patterns. The BO and RO models consistently demonstrate superior performance in accurately selecting “outstanding lines”.

Although the “O” method generally exhibits better performance, the “S” method stands out for its computational efficiency, especially evident in improving Sensitivity in BO and RO models. Furthermore, it is emphasized that, for genetic improvement, high values in Sensitivity, Kappa, and F1 are more desirable than in Specificity, aligning with the objective of effectively identifying the top lines. These results reflect the necessary balance that must exist between accuracy and efficiency in selecting “outstanding lines”.

### 3.2. Maize_2

**Figure 4:**
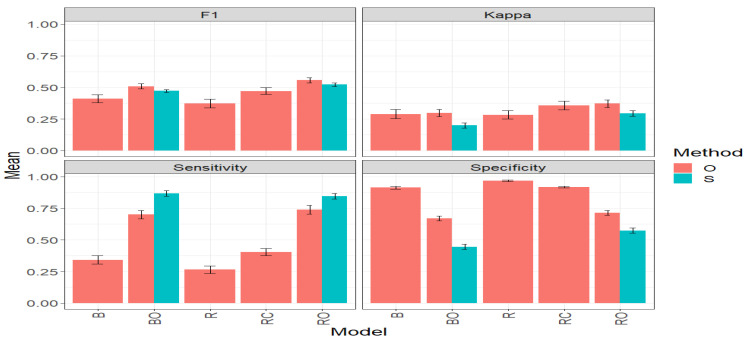
Prediction performance for dataset Maize_2 using original (O) and simplified (S) methods. The results are presented for models B, BO, R, RC, and RO in terms of the metrics: F1 score, Kappa coefficient, Sensitivity, and Specificity.

Figure 4 (Table A2, Appendix A) shows the comparison of the performance achieved by the “O” and “S” methods using the results obtained by the BO and RO models. It is observed that in terms of the F1 Score, the “O” methods outperforms the “S” methods in both models. For example, for the BO model, the average F1 Score value in the “O” method (0.5086) is 7.81% higher than in the “S” method (0.4717). In the case of the RO model, the average F1 Score value in the “O” method (0.5562) is 6.46% higher than in the “S” method (0.5224). However, it is noted that the difference between the models is not very high. Additionally, it should be considered that the S model has a simpler operation, requiring fewer computational requirements than the O model.

#### 3.2.1. F1 Score

Under the F1 Score metric, the RO model excels in the “O” method with an average value of 0.5562. It is closely followed by the BO model with a value of 0.5086, representing a significant increase of 23.84% compared to the B model, which had an average value of 0.4107. However, BO was surpassed by RO by 9.36%. In third place is the RC model with an F1 value of 0.4713, surpassing B by 14.76%. The R model exhibited the poorest performance with an average value of 0.3739, marking a significant decrease of 32.78% compared to RO and 26.49% compared to BO.

#### 3.2.2. Kappa Coefficient

Under the Kappa metric, in the “O” method, the RO model stands out with an average value of 0.3716. It is followed by the RC model with 0.3576, and then BO (0.2979), B (0.2885), and R (0.2828) in that order, as shown in Figure 4 and Table A2. According to these results, RO surpasses BO by 24.74%. Regarding the R model, which exhibits the poorest performance, it marks a significant decrease of 23.89% compared to RO.

When directly comparing the “O” and “S” methods using the BO and RO models, it is observed that, in terms of Kappa, the “O” method evidently outperforms the “S” method. For the BO case, the average Kappa value in the “O” method (0.2979) is 49.87% higher than in the “S” method (0.1988). For the RO model, the average Kappa value in the “O” method (0.3716) is 26.13% higher than in the “S” method (0.2946). Given these results, it is important to emphasize that before settling for a method, it is necessary to carefully review all the evaluated metrics.

#### 3.2.3. Sensitivity

Under this metric, the RO and BO models stand out significantly compared to the RC, B, and R models, under the “O” method. This is because RO and BO obtain the highest average Sensitivity values, 0.7398 and 0.7003, respectively. For example, for RO, this represents a significant increase of 179.65% compared to the R model, which had the lowest average value (0.2645) among all models. In the same vein, BO surpasses the R model by 164.74%.Thus, the difference between RO and BO is only 5.31%, with BO being slightly lower, placing both models as the best options in this method.

Finally, we compare the “O” and “S” methods using only the BO and RO models. In Figure 4, it is observed that, in terms of Sensitivity, the “S” method strongly surpasses the “O” method, which had not happened in the results of the F1 and Kappa metrics for the Maize_2 dataset. For BO, the average Sensitivity value in the “S” method (0.8687) is 24.03% higher than in the “O” method (0.7004). Similarly, for RO, the average Sensitivity value in the “S” method (0.8461) is 14.37% higher than in the “O” method (0.7398). These results highlight the importance of considering multiple studies before settling on a particular method and/or model.

#### 3.2.4. Specificity

The R model stands out with the highest average Specificity value (0.9681), indicating a strong ability to identify the “non-top” lines, for the “O” method. It is closely followed by the RC and B models with average values of 0.9182 and 0.9135, respectively. On the other hand, the lowest performances are obtained by the RO and BO models, with an average Specificity of 0.7148 and 0.6709, respectively. This indicates, for example, that BO has a 30.70% lower capacity to effectively identify the non-top lines compared to the R model. Remembering that achieving very high values of Specificity is not desirable, and it is better to excel in other metrics.

When comparing the “O” and “S” methods directly using the BO and RO models, it is observed that, in terms of Specificity, the “O” method outperforms the “S” method. For BO, the average Specificity value in the “O” method (0.6709) is 50.55% higher than in the “S” method (0.4456). For RO, the average Specificity value in the “O” method (0.7148) is 24.43% higher than in the “S” method (0.5745). Consequently, and remembering that a very high value of Specificity is not necessarily the best, as it affects Sensitivity, the S method exhibits better behavior.

In summary, this exhaustive study on the “O” and “S” methods applied to the Maize_2 dataset, evaluating crucial metrics such as F1, Kappa, Sensitivity, and Specificity, reveals clear patterns. The BO and RO models consistently demonstrate superior performance in the precise selection of outstanding lines. Although the “O” method generally exhibits better performance, the “S” method stands out for its computational efficiency, especially evident in improving the Sensitivity value in BO and RO models. Furthermore, it is emphasized that, for genetic improvement, high values in Sensitivity, Kappa, and F1 are more desirable than in Specificity, aligning with the objective of effectively identifying the top lines. These results reflect the necessary balance that must exist between Precision and efficiency in the selection of outstanding lines.

### 3.3. Maize_3

**Figure 5:**
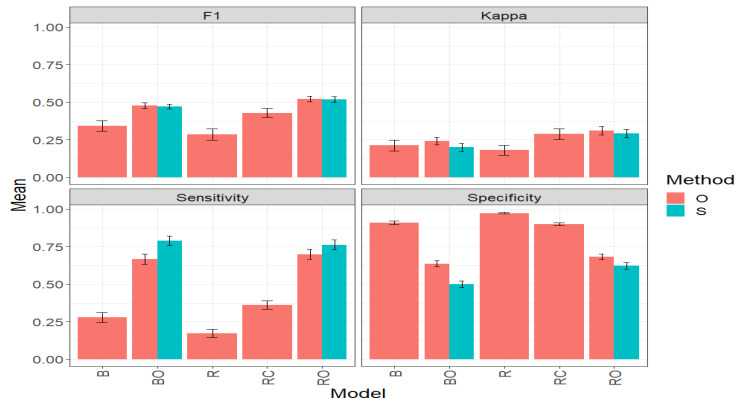
Prediction performance for dataset Maize_3 using original (O) and simplified (S) methods. The results are presented for models B, BO, R, RC, and RO in terms of the metrics: F1 score, Kappa coefficient, Sensitivity, and Specificity.

The obtained results, for the Maize:3 dataset, are presented in Figure 5 and Table A3 (Appendix A). The results are organized for each method, for each of the five models (B, BO, R, RC, and RO), and across the four classification metrics (F1, Kappa, Sensitivity, and Specificity).

#### 3.3.1. F1 Score

Under the F1 Score metric, the RO model stands out in the “O” method with an average value of 0.5214. It is closely followed by the BO model with a value of 0.4778, representing a significant increase of 38.93% compared to the B model, which had an average value of 0.3439. However, BO was surpassed by RO by 9.15%. In third place is the RC model with an F1 value of 0.4284, surpassing B by 24.58%. The R model exhibited the poorest performance with an average value of 0.2856, marking a significant decrease of 45.23% compared to RO, and 40.22% compared to BO.

When directly comparing the “O” and “S” methods using the BO and RO models, it is observed that in terms of F1 Score, both methods achieve very similar performance, slightly surpassed by the “O” method in both models. For BO, the average F1 Score value in the “O” method (0.4778) is 1.29% higher than in the “S” method (0.4716). For RO, the average F1 Score value in the “O” method (0.5214) is 0.5% higher than in the “S” method (0.5189). Besides this small difference, it should be considered that the S model has a simpler operation, demanding fewer computational requirements than the O model.

#### 3.3.2. Kappa Coefficient

Under the Kappa metric, in the “O” method, the RO model stands out with an average value of 0.3114. It is followed by the RC model with 0.2885, and then declining is the BO model (0.2413), B (0.2124), and R (0.1817), in that order. According to these results, RO surpasses BO by 29.05%. Regarding the R model, which exhibits the poorest performance, it marks a significant decrease of 41.67% compared to the RO model.

When comparing the “O” and “S” methods directly using the models BO and RO, it is observed that, in terms of Kappa, the method “O” surpasses the method “S”. For the case of BO, the average Kappa value in the “O” method (0.2413) is 20.89% higher than in the “S” method (0.1996). For the RO model, the average Kappa value in the “O” method (0.3114) is 6.42% higher than in the “S” method (0.2926). Given these results, it is important to emphasize that before settling for a method, it is necessary to carefully review all the evaluated metrics.

#### 3.3.3. Sensitivity

In the “O” method, the models RO and BO stand out significantly compared to the models RC, B, and R; since RO and BO obtain the highest average Sensitivity values of 0.6985 and 0.6657, respectively. This represents, for example, for RO, a significant increase of 302.0% compared to the R model, which had the lowest average value (0.1738) among all the models. In the case of the BO model, it surpasses the R model by 283.10%, very similar to RO’s performance.

When comparing the “O” and “S” methods directly using the models BO and RO, it is observed that, in terms of Sensitivity, the method “S” strongly outperforms the method “O”, which did not happen in the results of the F1 and Kappa metrics for the Maize_3 dataset. For BO, the average Sensitivity value in the “S” method (0.7884) is 18.43% higher than in the “O” method (0.6657). Likewise, for RO, the average Sensitivity value in the “S” method (0.7613) is 8.99% higher than in the “O” method (0.6986). These results highlight the importance of considering several studies before settling for a specific method and/or model.

#### 3.3.4. Specificity

In the “O” method, the R model stands out with the highest average Specificity value (0.9726), indicating a strong ability to identify the “non-top” lines. It is closely followed by the B and RC models with average values of 0.9074 and 0.8979, respectively. On the other hand, the RO and BO models show the lowest performances, with an average Specificity of 0.6828 and 0.6361, respectively. This indicates, for example, that BO has a 34.6% lower capacity to effectively identify the non-top lines compared to the R model.

When comparing the “O” and “S” methods directly using the models BO and RO, it is observed that, in terms of Specificity, the method “O” outperforms the method “S”. For BO, the average Specificity value in the “O” method (0.6361) is 27.47% higher than in the “S” method (0.4990). For RO, the average Specificity value in the “O” method (0.6828) is 9.74% higher than in the “S” method (0.6222). Consequently, and remembering that a very high value of Specificity is not necessarily the best, as it affects Sensitivity, the S method exhibits better behavior.

In summary, this comprehensive study on the “O” and “S” methods applied to the Maize_3 dataset, evaluating crucial metrics such as F1, Kappa, Sensitivity, and Specificity, reveals clear patterns. The models BO and RO consistently demonstrate superior performance in the precise selection of outstanding lines. Although the “O” method generally exhibits better performance, the “S” method stands out for its computational efficiency, especially evident in improving the value of Sensitivity in BO and RO models. Furthermore, it is emphasized that, for genetic improvement, high values in Sensitivity, Kappa, and F1 are more desirable than in Specificity, aligning with the objective of effectively identifying the top lines. These results reflect the necessary balance that must exist between Precision and efficiency in the selection of outstanding lines.

### 3.4. Soybean

**Figure 6:**
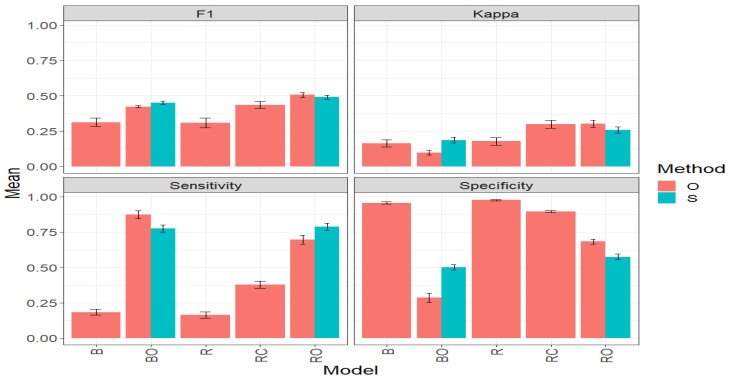
Prediction performance for dataset Soybean using Original (O) and simplified (S) methods. The results are presented for models B, BO, R, RC, and RO in terms of the metrics: F1 score, Kappa coefficient, Sensitivity, and Specificity.

The results for the Soybean dataset have been presented in Figure 6 and Table A4 (Appendix A). The arrangement of results has been structured, considering each method, the five distinct models (B, BO, R, RC, and RO), and the four crucial classification metrics (F1, Kappa, Sensitivity, and Specificity).

#### 3.4.1. F1 Score

Under the F1 Score, the RO model emerges as the leader in the “O” method with a prominent average value of 0.5055. It is closely followed by the RC model, registering a value of 0.4335, representing a notable increase of 38.66% compared to the B model, which averaged 0.3126. However, RC was surpassed by RO by 16.62%. In third place is the BO model, with an F1 value of 0.4226, surpassing B by 35.17%. The R model exhibited the lowest performance with an average value of 0.3083, marking a substantial decrease of 63.96% compared to RO and 27.03% compared to BO.

When directly comparing the “O” and “S” approaches applying the BO and RO models, it becomes evident that in terms of the F1 Score, both methods achieve very similar performance. For BO, the average F1 Score value in the “S” approach (0.4505) surpasses by 6.61% that obtained in the “O” approach (0.4226). In contrast, for RO, the average F1 Score value in the “O” approach (0.5055) is 3.02% higher than in the “S” approach (0.4907). In addition to this slight difference, it is crucial to consider that the S model is characterized by a simpler operation, requiring fewer computational capabilities compared to the O model.

#### 3.4.2. Kappa Coefficient

Under the Kappa criteria, in the “O” method, the RO model stands out with an average value of 0.3025. It is closely followed by the RC model with 0.2993, followed by the R model (0.1778), B (0.1636), and BO (0.0967), in that order, showing a marked decline in performance. With these results, RO surpasses RC by 1.07%. In contrast, the BO model exhibits the worst performance, with a significant decrease of 68.05% compared to the RO model.

When contrasting the “O” and “S” methods directly using the BO and RO models, a balance in terms of Kappa is evident. For BO, the average Kappa value in the “S” approach (0.1871) is notably higher, with an increase of 93.62% compared to the “O” approach (0.0966). On the other hand, for RO, the average Kappa value in the “O” approach (0.3025) is higher, with an increase of 17.49%, compared to the value in the “S” approach (0.2574). These findings emphasize the importance of carefully evaluating all metrics before deciding on the most appropriate method.

#### 3.4.3. Sensitivity

In the “O” method, the BO and RO models stand out significantly compared to the RC, B, and R models. BO and RO achieve the highest average Sensitivity values, with 0.8724 and 0.6956, respectively. This represents, for example, in the case of BO, a significant increase of 436.49% compared to the R model, which obtained the lowest average value (0.1626) among all the models. Likewise, the RO model surpasses the R model by 327.75%, showing performance close to that of BO.

When making a direct comparison between the “O” and “S” methods using the BO and RO models, a balance in terms of Sensitivity is evident. For BO, the average Sensitivity value in the “O” method (0.8724) is higher by 12.61% than the value in the “S” method (0.7747). On the other hand, for RO, the average Sensitivity value in the “S” method (0.7882) is 13.32% higher than in the “O” method (0.6956). These results underscore the importance of considering multiple studies before deciding on a specific method and/or model.

#### 3.4.4. Specificity

In the “O” approach, the R model stands out with the highest average Specificity value, reaching 0.9756, indicating its strong ability to identify the “non-top” lines. Subsequently, B and RC follow with the best performances, with average values of 0.9549 and 0.8947, respectively. In contrast, the RO and BO models show the lowest performances, with an average Specificity of 0.6826 and 0.2851, respectively. This highlights, for example, that BO has a 70.78% lower ability to effectively identify the “non-top” lines compared to the R model.

**Figure 7:**
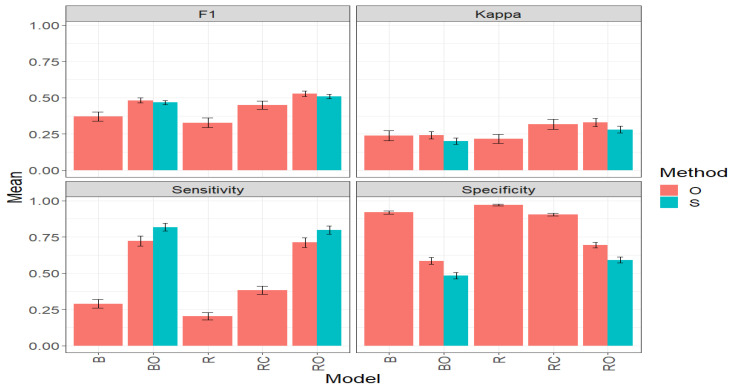
Prediction performance for Across_Data using original (O) and simplified (S) methods. The results are presented for models B, BO, R, RC, and RO in terms of the metrics: F1 score, Kappa coefficient, Sensitivity, and Specificity.

The results of comparing the “O” and “S” methods using the models BO and RO demonstrate a balance in terms of Specificity, as shown in Figure 7 and Table A5 (see Appendix A). For BO, the average Specificity value in the “S” method (0.5001) significantly surpasses by 75.64% the value in the “O” method (0.2851). On the other hand, for RO, the average Specificity value in the “O” method (0.6826) is 18.91% higher than in the “S” method (0.5740). Consequently, and bearing in mind that a very high value of Specificity is not always the best, as it can affect Sensitivity, the S method shows a better behavior in terms of RO, while the O method stands out in terms of BO.

In summary, this exhaustive study of the “O” and “S” methods applied to the Soybean dataset, evaluating crucial metrics such as F1, Kappa, Sensitivity, and Specificity, reveals clear patterns. The BO and RO models consistently demonstrate superior performance in the precise selection of “outstanding lines”. Although the “O” method generally exhibits better performance, the “S” method stands out for its computational efficiency, especially evident in improving the Sensitivity value in BO and RO models. Furthermore, it is emphasized that, for genetic improvement, high values in Sensitivity, Kappa, and F1 are more desirable than in Specificity, aligning with the objective of effectively identifying the top lines. These results reflect the necessary balance that must exist between Precision and efficiency in the selection of “outstanding lines”.

### 3.5. Across_Data

Figure 7 and Table A5 (Appendix A) present the average results for Across_Data. The Across_Data was computed by averaging the results of all the datasets with the goal of obtaining a complete picture of the prediction performance of the proposed methods.

#### 3.5.1. F1 Score

Under the F1 Score metric, it is observed that the model RO stands out in the “O” method with an average value of 0.5276. It is closely followed by the BO model with a value of 0.4813, representing a significant increase of 29.88% compared to model B, which had an average value of 0.3706. However, BO is surpassed by RO by 9.62%. In third place is the RC model with an F1 value of 0.4485, surpassing B by 21.03%. The R model shows the worst performance with an average value of 0.3279, marking a significant decrease of 37.84% compared to RO and 31.86% compared to BO.

In the “S” method, the RO model again stands out with an average F1 Score value of 0.5097. The BO model comes second with an average value of 0.4676. It is closely followed by the RC model with a value of 0.4533, representing a reduction of 3.07% compared to the performance achieved by BO. Additionally, RO surpasses RC by 12.45% in the ability to select the top lines of the dataset. The lowest results are presented by the B and R models, with R showing the worst performance with an average value of 0.3297. Therefore, RO and BO achieve an improvement of 54.60% and 41.83% compared to the R model, respectively.

Comparing the “O” and “S” methods directly using the BO and RO models, it is observed that in terms of F1 Score, both methods achieve very similar performance, superior in the “O” method for both models. For BO, the average F1 Score value in the “O” method (0.4813) is 2.97% higher than in the “S” method (0.4676). For RO, the average F1 Score value in the “O” method (0.5276) is 3.51% higher than in the “S” method (0.5097). Furthermore, this small difference should be considered in light of the fact that the S model has a simpler operation, demanding fewer computational requirements than the O model.

#### 3.5.2. Kappa Coefficient

Under the Kappa metric, in the “O” method, the model RO stands out with an average value of 0.3289. It is followed by the RC model with 0.3164, and then descending the BO model (0.2415), B (0.2393), and R (0.2161), in that order. According to these results, RO surpasses BO by 36.21%. Regarding the R model, which shows the worst performance, it marks a significant decrease of 34.29% compared to the RO model.

Comparing the “O” and “S” methods directly using the BO and RO models, it is observed that, in terms of Kappa, the “O” method surpasses the “S” method. For BO, the average Kappa value in the “O” method (0.2415) is 20.24% higher than in the “S” method (0.2008). For the RO model, the average Kappa value in the “O” method (0.3289) is 17.05% higher than in the “S” method (0.2809). Given these results, it is important to emphasize that before choosing a method, it is necessary to carefully review all the evaluated metrics.

#### 3.5.3. Sensitivity

In the “O” method, the BO and RO models stand out significantly compared to the RC, B, and R models; as BO and RO achieve the highest average Sensitivity values of 0.7224 and 0.7117, respectively. This represents, for example, a significant increase of 255.68% for BO compared to the R model, which had the lowest average value (0.2031) among all models. In the case of the BO model, it surpasses the R model by 250.41%, very similar to RO’s performance.

Comparing the “O” and “S” methods directly using the BO and RO models, it is observed that, in terms of Sensitivity, the “S” method strongly surpasses the “O” method, which does not occur in the F1 and Kappa metric results for the Across_Data dataset. For BO, the average Sensitivity value in the “S” method (0.8182) is 13.27% higher than in the “O” method (0.7224). Likewise, for RO, the average Sensitivity value in the “S” method (0.7983) is 12.8% higher than in the “O” method (0.7117). These results underscore the importance of considering various studies before opting for a particular method and/or model.

#### 3.5.4. Specificity

In the “O” method, the R model stands out with the highest average Specificity value (0.9718), indicating a strong ability to identify the “non-top” lines. It is closely followed by the B and RC models with average values of 0.9199 and 0.9044, respectively. On the other hand, the RO and BO models show the lowest Specificity performances, with an average Specificity of 0.6944 and 0.5858, respectively. This indicates, for example, that BO and RO have a 39.72% and 28.54% lower ability to effectively identify the “non-top” lines compared to the R model, respectively.

Comparing the “O” and “S” methods directly using the BO and RO models, it is observed that, in terms of Specificity, the “O” method surpasses the “S” method. For BO, the average Specificity value in the “O” method (0.5858) is 20.86% higher than in the “S” method (0.4848). For RO, the average Specificity value in the “O” method (0.6944) is 17.39% higher than in the “S” method (0.5915). Consequently, and bearing in mind that a very high value of Specificity is not necessarily the best, as it affects Sensitivity, the “S” method exhibits better behavior.

In summary, this comprehensive study evaluated the performance of different models and methods across datasets using fundamental metrics such as F1 Score, Kappa, Sensitivity, and Specificity. The results reveal that, in terms of the F1 Score, the model RO consistently shows superior performance in the “O” method, closely followed by the BO model. Although the “O” method has an advantage in the F1 Score, the “S” method stands out for its computational efficiency and improvement in Sensitivity for BO and RO. Under Kappa, RO also excels in both methods, showcasing its robustness. In Sensitivity, BO and RO remarkably stand out in the “S” method, vastly surpassing the “O” method. On the other hand, in Specificity, the “O” method exhibits superior performance over the “S” method. However, it is emphasized that for genetic improvement, high values in Sensitivity, Kappa, and F1 are more desirable than in Specificity, aligning to effectively identify top lines. These findings underscore the importance of considering multiple metrics and methods when choosing the optimal approach for selecting outstanding lines.

## 4. Discussion

Achieving high prediction accuracies in the application of the GS methodology necessitates several prerequisites; yet, efficiently optimizing the numerous factors that influence its accuracy poses a significant challenge. Given the importance of enhancing predictive methodologies, considerable research efforts are directed towards identifying and implementing strategies that can significantly improve its efficiency. Various studies have studied the impact of the size of the training set and diversity on the accuracy of these methods. Furthermore, investigations have explored how the population structure and its genetic relationship with the breeding population influence the Precision of genomic prediction. Other research areas include the effects of marker density and distribution, linkage disequilibrium, the genetic architecture and heritability of traits, and the exploration of novel statistical machine learning models [5]. Additionally, the integration of supplementary inputs such as proteomics, metabolomics, and enviromics has been examined for their potential to enrich and refine predictive analytics. All these investigations try to improve the efficiency of the GS methodology and show that improving the GS methodology is an ongoing process that requires a combination of innovative methodologies, rigorous validation, and a deep understanding of the biological context. Additionally, staying informed about emerging technologies and ethical considerations is essential in this field.

Regarding statistical machine learning methods, many parametric (mixed models, Bayesian methods) and nonparametric (deep learning, random forest, gradient boosting machines, etc.) state-of-the-art algorithms have been explored in the context of genomic prediction. Some notable algorithms that aim to leverage genetic data to predict various phenotypic traits or outcomes are GBLUP, Bayesian methods (A, B, C, Lasso, etc), random forest, gradient boosting machine, support vector machine, deep learning, kernel methods, etc. However, the predictions resulting from most of these algorithms are not yet optimal for practical applications. For these reasons, researchers continue to work on improving genomic prediction models by developing more sophisticated algorithms, incorporating additional sources of data (e.g., transcriptomics, epigenetics), and refining methods for accounting for complex genetic interactions and environmental factors. While these models have made significant progress, achieving optimal accuracy in all scenarios remains a complex and ongoing challenge in genomics.

The genomic prediction models, while providing extensive results, were validated by comparing predicted values with observed phenotypic data. The high agreement between observed and predicted values supports the practical applicability of these models in breeding programs. Furthermore, cross-validation across environments demonstrated the models’ robustness and generalizability, making them useful in predicting traits under various environmental conditions, even those not represented in the original dataset.

These findings suggest that while the statistical methods are sophisticated, their practical value lies in their ability to reduce the need for exhaustive field testing, accelerating the selection process by enabling breeders to focus on high-potential genotypes early in the breeding cycle. This application demonstrates that genomic prediction can be an effective tool for enhancing breeding efficiency and genetic gain across diverse environments.

Therefore, intending to enhance the accuracy in identifying the superior (or inferior) lines, this research delineates and contrasts five established methodologies for selecting the top (or bottom) lines evaluating the prediction accuracy in terms of novel metrics popular in the context like Sensitivity and Specificity. The description of each method was achieved with details to avoid any confusion between these methods and to see the simplicity and complexity of each one of them. In our comparative analysis with the five real datasets, we observed that under the original method, model RO outperformed the others in terms of F1 score. Specifically, it exceeded model B by 42.37%, model BO by 9.62%, model R by 60.87%, and model RC by 17.63%. Meanwhile, in terms of the Kappa coefficient, the RO model was superior to models B, BO, R, and RC by 37.46%, 36.21%, 52.18%, and 3.95%, respectively. In terms of Sensitivity, model RO outperformed models B, R, and RC by 145.74%, 250.41%, and 86.20%, respectively. Also, our results show that the second-best model was the BO, that only slightly worse than the RO model.

Our results unequivocally highlight the effectiveness of methods RO and BO in identifying the top lines. These methods incorporate a post-processing step to ensure comparable Sensitivity and Specificity, making them better choices. Nonetheless, it is important to acknowledge that achieving this enhanced classification accuracy comes at the cost of increased computational resources during the tuning process, specifically in the selection of the optimal threshold for final line classification. However, this upsurge in computational demands poses no significant challenge when dealing with small to moderately sized datasets. In such cases, only a single hyperparameter—the optimal threshold—needs tuning. Consequently, the advantages offered by the RO and BO methods far outweigh the associated costs. Furthermore, our research reveals that simplified versions of the original RO and BO methods remain highly competitive. These simplified approaches deliver nearly equivalent prediction performance while substantially reducing computational resource requirements compared to the original RO and BO methods. Given this finding, we encourage the adoption of the original RO and BO methods in real-world applications, especially for datasets of small to moderate size, where their implementation is straightforward and beneficial.

Additionally, our results clearly show that the R method consistently yields the lowest performance across all measures. This can be attributed to the persistent bias in predicting lines in the tails, whether they are top or bottom lines. Consequently, the R method tends to underestimate predictions for the top lines and overestimate predictions for the bottom lines, resulting in a significant misclassification error when selecting the optimal lines regarding a threshold.

Additionally, this paper offers a compelling perspective by conceptualizing the challenge of choosing the top (or bottom) lines in breeding programs as a classification problem. Even though some of the proposed methods (R, RC, and RO) employ a regression model during the initial training phase, this unique approach reframes the selection process as a classification problem. Consequently, the paper introduces a set of classification metrics, including Sensitivity, Specificity, F1 Score, and Kappa Coefficient, to assess the accuracy and quality of top line selection. These metrics provide a more appropriate and insightful means of evaluating the effectiveness of the chosen top lines. Also, our results show evidence that models BO and RO provide more balanced Sensitivity and Specificity regarding the other methods which is of paramount importance since metrics like Sensitivity and Specificity in plant breeding facilitate the precise selection of superior lines, optimization of breeding programs, reduction in errors, improvement of trait selection, and quantitative evaluation of line performance. These metrics play a crucial role in enhancing the efficiency, effectiveness, and success of plant breeding efforts.

### Assessing Sensitivity and Specificity

Sensitivity, defined as the model’s ability to correctly identify true positives, is a crucial metric in our analysis, particularly in the context of genomic prediction for breeding. However, we acknowledge that an emphasis on maximizing Sensitivity may inadvertently lead to an increase in false positives, which can complicate the selection of superior phenotypes.

To address this concern, we implemented several strategies to balance Sensitivity and Specificity within our models. We carefully selected thresholds for predicting positive outcomes that aim to minimize false positives while still capturing a high proportion of true positives. Additionally, we utilized cross-validation techniques to assess model performance across diverse datasets, ensuring that our findings are robust and generalizable.

Moreover, we conducted an analysis of the trade-offs involved in adjusting Sensitivity levels, discussing the implications for breeding programs. While higher Sensitivity is desirable to ensure that few superior phenotypes are missed, it is critical to monitor the rate of false positives to avoid misdirecting breeding efforts. This balanced approach allows us to enhance the Precision of our genomic predictions and improve the overall selection process.

These results offer empirical confirmation that the RC model, which represents the conventional approach for selecting the top lines, ranks among the two least efficient strategies in capturing top lines effectively in terms of Sensitivity. Consequently, we strongly advocate for the adoption of methods RO and BO by breeders. These methods have demonstrated their ability to enhance the efficacy of the Genomic Selection (GS) methodology. As previously noted, the practical implementation of GS remains challenging due to the influence of various factors on its performance. Therefore, embracing the RO and BO methods can significantly improve the overall results and reliability of GS in breeding programs.

## 5. Conclusions

In this paper, we described and evaluated five existing methods to select the top (or bottom) lines in the context of genomic prediction. We described each of the five existing methods simply and clearly with the goal that breeders and scientists of related fields can distinguish without any ambiguity the peculiarities of each method. Then, we evaluated the performance of the five methods with five real datasets for selecting the top lines using four popular metrics in the context of classification methods (F1 Score, Kappa coefficient, Sensitivity, and Specificity). We found that the methods BO and RO performed effectively across the five datasets for selecting the top lines with more accuracy. Methods BO and RO were better across traits, datasets, and environments than the other three methods in terms of F1 score, Kappa coefficient, and Sensitivity. For these reasons, we encourage other studies to increase the empirical evidence of the superiority of methods BO and RO to select the top (or bottom) lines.

## Figures and Tables

**Figure A1:**
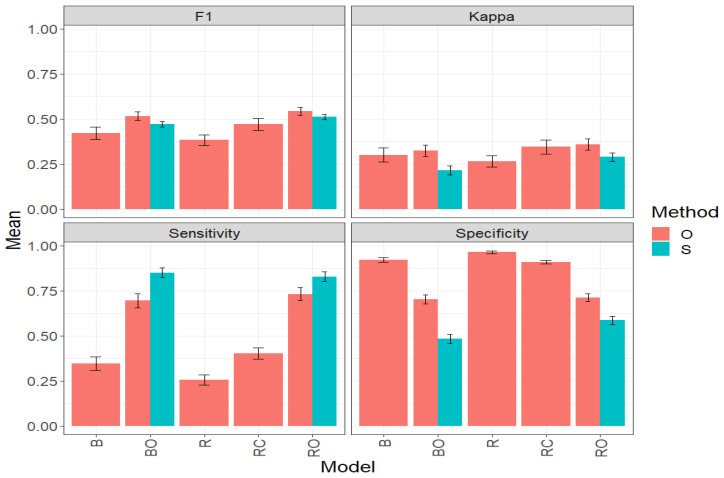
Prediction performance for dataset Maize_4 using original (O) and simplified (S) methods. The results are presented for models B, BO, R, RC, and RO in terms of the metrics: F1 score, Kappa coefficient, Sensitivity, and Specificity.

## Data Availability

Phenotypes along with genotypes are available through [21] [Alencar, X. (2021)]. Technical nuances of machine learning: implementation and validation of supervised methods for genomic prediction in plant breeding. Crop Breeding and Applied Biotechnology, 21(S): e381421S2. https://doi.org/10.1590/1984-70332021v21Sa15 used by Montesinos-Lopez et al. [14].

## Appendix A

**Table A1:** Comparison of models (B, BO, R, RC, and RO) under methods (S and O) for selecting the best candidates’ genotypes (Dataset: Maize_1) based on F1 Score, Kappa Coefficient, Sensitivity, and Specificity.

Dataset	Method	Model	Metric	Mean	SE
Maize_1	O	B	F1	0.364	0.033
Maize_1	O	BO	F1	0.481	0.017
Maize_1	O	R	F1	0.290	0.028
Maize_1	O	RC	F1	0.439	0.027
Maize_1	O	RO	F1	0.513	0.019
Maize_1	O	B	Kappa	0.231	0.035
Maize_1	O	BO	Kappa	0.246	0.024
Maize_1	O	R	Kappa	0.173	0.026
Maize_1	O	RC	Kappa	0.291	0.032
Maize_1	O	RO	Kappa	0.300	0.026
Maize_1	O	B	Sensitivity	0.298	0.032
Maize_1	O	BO	Sensitivity	0.678	0.033
Maize_1	O	R	Sensitivity	0.159	0.022
Maize_1	O	RC	Sensitivity	0.364	0.025
Maize_1	O	RO	Sensitivity	0.694	0.036
Maize_1	O	B	Specificity	0.904	0.012
Maize_1	O	BO	Specificity	0.634	0.020
Maize_1	O	R	Specificity	0.979	0.005
Maize_1	O	RC	Specificity	0.902	0.009
Maize_1	O	RO	Specificity	0.680	0.021
Maize_1	S	BO	F1	0.474	0.013
Maize_1	S	RO	F1	0.506	0.016
Maize_1	S	BO	Kappa	0.205	0.022
Maize_1	S	RO	Kappa	0.271	0.025
Maize_1	S	BO	Sensitivity	0.809	0.026
Maize_1	S	RO	Sensitivity	0.767	0.030
Maize_1	S	BO	Specificity	0.494	0.023
Maize_1	S	RO	Specificity	0.602	0.021

**Table A2:** Comparison of models (B, BO, R, RC, and RO) under methods (S and O) for selecting the best candidates’ genotypes (Dataset: Maize_2) based on F1 Score, Kappa Coefficient, Sensitivity, and Specificity.

Dataset	Method	Model	Metric	Mean	SE
Maize_2	O	B	F1	0.411	0.033
Maize_2	O	BO	F1	0.509	0.020
Maize_2	O	R	F1	0.374	0.034
Maize_2	O	RC	F1	0.471	0.028
Maize_2	O	RO	F1	0.556	0.021
Maize_2	O	B	Kappa	0.289	0.036
Maize_2	O	BO	Kappa	0.298	0.028
Maize_2	O	R	Kappa	0.283	0.033
Maize_2	O	RC	Kappa	0.358	0.033
Maize_2	O	RO	Kappa	0.372	0.030
Maize_2	O	B	Sensitivity	0.342	0.032
Maize_2	O	BO	Sensitivity	0.700	0.034
Maize_2	O	R	Sensitivity	0.265	0.029
Maize_2	O	RC	Sensitivity	0.405	0.028
Maize_2	O	RO	Sensitivity	0.740	0.034
Maize_2	O	B	Specificity	0.914	0.012
Maize_2	O	BO	Specificity	0.671	0.020
Maize_2	O	R	Specificity	0.968	0.007
Maize_2	O	RC	Specificity	0.918	0.008
Maize_2	O	RO	Specificity	0.715	0.019
Maize_2	S	BO	F1	0.472	0.012
Maize_2	S	RO	F1	0.522	0.014
Maize_2	S	BO	Kappa	0.199	0.019
Maize_2	S	RO	Kappa	0.295	0.023
Maize_2	S	BO	Sensitivity	0.869	0.021
Maize_2	S	RO	Sensitivity	0.846	0.023
Maize_2	S	BO	Specificity	0.446	0.020
Maize_2	S	RO	Specificity	0.574	0.020

**Table A3:** Comparison of models (B, BO, R, RC, and RO) under methods (S and O) for selecting the best candidates’ genotypes (Dataset: Maize_3) based on F1 Score, Kappa Coefficient, Sensitivity, and Specificity.

Dataset	Method	Model	Metric	Mean	SE
Maize_3	O	B	F1	0.344	0.034
Maize_3	O	BO	F1	0.478	0.018
Maize_3	O	R	F1	0.286	0.038
Maize_3	O	RC	F1	0.428	0.030
Maize_3	O	RO	F1	0.521	0.020
Maize_3	O	B	Kappa	0.212	0.037
Maize_3	O	BO	Kappa	0.241	0.026
Maize_3	O	R	Kappa	0.182	0.033
Maize_3	O	RC	Kappa	0.289	0.036
Maize_3	O	RO	Kappa	0.311	0.029
Maize_3	O	B	Sensitivity	0.278	0.033
Maize_3	O	BO	Sensitivity	0.666	0.034
Maize_3	O	R	Sensitivity	0.174	0.028
Maize_3	O	RC	Sensitivity	0.362	0.029
Maize_3	O	RO	Sensitivity	0.699	0.034
Maize_3	O	B	Specificity	0.907	0.013
Maize_3	O	BO	Specificity	0.636	0.019
Maize_3	O	R	Specificity	0.973	0.005
Maize_3	O	RC	Specificity	0.898	0.009
Maize_3	O	RO	Specificity	0.683	0.019
Maize_3	S	BO	F1	0.472	0.016
Maize_3	S	RO	F1	0.519	0.019
Maize_3	S	BO	Kappa	0.200	0.026
Maize_3	S	RO	Kappa	0.293	0.028
Maize_3	S	BO	Sensitivity	0.788	0.031
Maize_3	S	RO	Sensitivity	0.761	0.033
Maize_3	S	BO	Specificity	0.499	0.022
Maize_3	S	RO	Specificity	0.622	0.022

**Table A4:** Comparison of models (B, BO, R, RC, and RO) under methods (S and O) for selecting the best candidates’ genotypes (Dataset: Soybean) based on F1 Score, Kappa Coefficient, Sensitivity, and Specificity.

Dataset	Method	Model	Metric	Mean	SE
Soybean	O	B	F1	0.313	0.029
Soybean	O	BO	F1	0.423	0.008
Soybean	O	R	F1	0.308	0.034
Soybean	O	RC	F1	0.433	0.025
Soybean	O	RO	F1	0.506	0.017
Soybean	O	B	Kappa	0.164	0.024
Soybean	O	BO	Kappa	0.097	0.015
Soybean	O	R	Kappa	0.178	0.028
Soybean	O	RC	Kappa	0.299	0.030
Soybean	O	RO	Kappa	0.302	0.024
Soybean	O	B	Sensitivity	0.183	0.021
Soybean	O	BO	Sensitivity	0.872	0.028
Soybean	O	R	Sensitivity	0.163	0.023
Soybean	O	RC	Sensitivity	0.378	0.025
Soybean	O	RO	Sensitivity	0.696	0.031
Soybean	O	B	Specificity	0.955	0.008
Soybean	O	BO	Specificity	0.285	0.033
Soybean	O	R	Specificity	0.976	0.005
Soybean	O	RC	Specificity	0.895	0.007
Soybean	O	RO	Specificity	0.683	0.017
Soybean	S	BO	F1	0.451	0.013
Soybean	S	RO	F1	0.491	0.014
Soybean	S	BO	Kappa	0.187	0.019
Soybean	S	RO	Kappa	0.257	0.021
Soybean	S	BO	Sensitivity	0.775	0.027
Soybean	S	RO	Sensitivity	0.788	0.025
Soybean	S	BO	Specificity	0.501	0.020
Soybean	S	RO	Specificity	0.574	0.020

**Table A5:** Comparison of models (B, BO, R, RC, and RO) under methods (S and O) for selecting the best candidates’ genotypes (Dataset: Across_Data) based on F1 Score, Kappa Coefficient, Sensitivity, and Specificity.

Dataset	Method	Model	Metric	Mean	SE
Across_data	O	B	F1	0.371	0.033
Across_data	O	BO	F1	0.481	0.017
Across_data	O	R	F1	0.328	0.033
Across_data	O	RC	F1	0.449	0.029
Across_data	O	RO	F1	0.528	0.020
Across_data	O	B	Kappa	0.239	0.034
Across_data	O	BO	Kappa	0.241	0.025
Across_data	O	R	Kappa	0.216	0.031
Across_data	O	RC	Kappa	0.316	0.034
Across_data	O	RO	Kappa	0.329	0.028
Across_data	O	B	Sensitivity	0.290	0.031
Across_data	O	BO	Sensitivity	0.722	0.034
Across_data	O	R	Sensitivity	0.203	0.026
Across_data	O	RC	Sensitivity	0.382	0.028
Across_data	O	RO	Sensitivity	0.712	0.034
Across_data	O	B	Specificity	0.920	0.012
Across_data	O	BO	Specificity	0.586	0.024
Across_data	O	R	Specificity	0.972	0.006
Across_data	O	RC	Specificity	0.904	0.008
Across_data	O	RO	Specificity	0.694	0.019
Across_data	S	BO	F1	0.468	0.014
Across_data	S	RO	F1	0.510	0.016
Across_data	S	BO	Kappa	0.201	0.022
Across_data	S	RO	Kappa	0.281	0.024
Across_data	S	BO	Sensitivity	0.818	0.026
Across_data	S	RO	Sensitivity	0.798	0.027
Across_data	S	BO	Specificity	0.485	0.022
Across_data	S	RO	Specificity	0.592	0.021

## Appendix B

### Appendix B.1. Maize_4 Data

For the Maize_4 dataset, the obtained results are illustrated in Figure A1 and detailed in Table A6 (Appendix B). The results are organized for each method and each of the five models (B, BO, R, RC, and RO), and are presented based on the four classification metrics (F1, Kappa, Sensitivity, and Specificity).

### Appendix B.2. F1 Score

Regarding the F1 Score, the RO model stands out in the “O” method with an average value of 0.5424. It is closely followed by the BO model with a value of 0.5165, showing a significant increase of 22.38% compared to the B model, which had an average value of 0.4220. However, RO surpasses BO by 5.03%. In third place, we find the RC model with an F1 value of 0.4705, surpassing B by 11.49%. The R model showed the poorest performance with an average value of 0.3826, marking a significant decrease of 29.47% compared to RO and 25.92% compared to BO.

When directly comparing the “O” and “S” methods using the BO and RO models, it is observed that in terms of F1 Score, both methods achieve very similar performance, with the “O” method being slightly superior in both models. For BO, the average F1 Score value in the “O” method (0.5165) is 9.79% higher than in the “S” method (0.4704). For RO, the average F1 Score value in the “O” method (0.5424) is 6.13% higher than in the “S” method (0.5111). Additionally, it is important to consider that the S model has a simpler operation, demanding fewer computational resources than the O model.

### Appendix B.3. Kappa Coefficient

In terms of Kappa, in the “O” method, the RO model stands out with an average value of 0.3587. It is followed by the RC model with 0.3453, and then decreasingly, the BO model (0.3253), B (0.3005), and R (0.2650), in that order. According to these results, RO surpasses BO by 10.26%. Regarding the R model, which shows the poorest performance, a significant decrease of 26.11% is observed compared to the RO model.

When comparing the “O” and “S” methods directly using the models BO and RO, it is observed that in terms of Kappa, the “O” method surpasses the “S” method. For BO, the average Kappa value in the “O” method (0.3553) is 52.08% higher than in the “S” method (0.2139). For the RO model, the average Kappa value in the “O” method (0. 3587) is 19.48% higher than in the “S” method (0.2888). In light of these results, it is crucial to emphasize that before opting for a method, it is necessary to carefully review all the evaluated metrics.

### Appendix B.4. Sensitivity

Regarding the Sensitivity metric, in the “O” method, the RO and BO models stand out significantly compared to the RC, B, and R models, as RO and BO obtain the highest average Sensitivity values, with 0.7308 and 0.6949, respectively. This represents, for example, in the case of RO, a significant increase of 185.56% compared to the R model, which had the lowest average value (0.2559) among all the models. In the case of the BO model, it surpasses the R model by 171.55%, showing a performance very similar to RO.

When comparing the “O” and “S” methods directly using the models BO and RO, it is observed that in terms of Sensitivity, the “S” method clearly surpasses the “O” method, which was not reflected in the results of the F1 and Kappa metrics for the Maize_4 dataset. For BO, the average Sensitivity value in the “O” method (0.7029) is 45.15% higher than in the “S” method (0.4842). For RO, the average Sensitivity value in the “O” method (0.7120) is 21.64% higher than in the “S” method (0.5854). However, it is fundamental to remember that an excessively high value of Specificity does not necessarily represent the best, as it can affect Sensitivity. In this regard, the S method exhibits more balanced behavior.

In summary, this comprehensive study on the “O” and “S” methods applied to the Maize_4 dataset, evaluating crucial metrics such as F1, Kappa, Sensitivity, and Specificity, reveals clear patterns. The models BO and RO consistently demonstrate superior performance in the precise selection of “outstanding lines”. Although the “O” method generally exhibits slightly better performance, the “S” method stands out for its computational efficiency, particularly evident in improving the value of Sensitivity in BO and RO models. Furthermore, it is emphasized that, for genetic improvement, high values in Sensitivity, Kappa, and F1 score are more desirable than in Specificity, aligning with the objective of effectively identifying the top lines.

**Table A6:** Comparison of models (B, BO, R, RC, and RO) under methods (S and O) for selecting the best candidates genotypes (Dataset: Maize_4 data) based on F1 Score, Kappa Coefficient, Sensitivity, and Specificity.

Dataset	Method	Model	Metric	Mean	SE
Maize_4	O	B	F1	0.422	0.034
Maize_4	O	BO	F1	0.516	0.023
Maize_4	O	R	F1	0.383	0.029
Maize_4	O	RC	F1	0.471	0.032
Maize_4	O	RO	F1	0.542	0.022
Maize_4	O	B	Kappa	0.301	0.039
Maize_4	O	BO	Kappa	0.325	0.031
Maize_4	O	R	Kappa	0.265	0.032
Maize_4	O	RC	Kappa	0.345	0.040
Maize_4	O	RO	Kappa	0.359	0.031
Maize_4	O	B	Sensitivity	0.346	0.036
Maize_4	O	BO	Sensitivity	0.695	0.040
Maize_4	O	R	Sensitivity	0.256	0.027
Maize_4	O	RC	Sensitivity	0.403	0.032
Maize_4	O	RO	Sensitivity	0.731	0.036
Maize_4	O	B	Specificity	0.920	0.013
Maize_4	O	BO	Specificity	0.703	0.026
Maize_4	O	R	Specificity	0.964	0.008
Maize_4	O	RC	Specificity	0.910	0.009
Maize_4	O	RO	Specificity	0.712	0.021
Maize_4	S	BO	F1	0.470	0.016
Maize_4	S	RO	F1	0.511	0.015
Maize_4	S	BO	Kappa	0.214	0.025
Maize_4	S	RO	Kappa	0.289	0.023
Maize_4	S	BO	Sensitivity	0.850	0.026
Maize_4	S	RO	Sensitivity	0.829	0.025
Maize_4	S	BO	Specificity	0.484	0.025
Maize_4	S	RO	Specificity	0.585	0.024
